# A novel method for delineation of oral mucosa for radiotherapy dose–response studies

**DOI:** 10.1016/j.radonc.2015.02.020

**Published:** 2015-04

**Authors:** Jamie A. Dean, Liam C. Welsh, Sarah L. Gulliford, Kevin J. Harrington, Christopher M. Nutting

**Affiliations:** a Joint Department of Physics at The Institute of Cancer Research and The Royal Marsden NHS Foundation Trust, London, UK; b Head and Neck Unit, The Royal Marsden NHS Foundation Trust, London, UK

**Keywords:** Contouring, Delineation, Oral mucosa, Oral mucositis, Dose–response

## Abstract

There is currently no standard method for delineating the oral mucosa and most attempts are oversimplified. A new method to obtain anatomically accurate contours of the oral mucosa surfaces was developed and applied to 11 patients. This is expected to represent an opportunity for improved toxicity modelling of oral mucositis.

Mucositis of the oral mucosa is a common and important acute toxicity in head and neck radiotherapy warranting efforts to mitigate its severity and impact on patients. It may result in reduced quality of life due to pain, dysphagia [1], [2], [3], weight loss and aspiration [4], [5]. These effects can result in patients being hospitalised and missing treatment fractions [6], compromising locoregional control [7], [8]. Mucositis is also frequently the dose-limiting toxicity in dose escalation and accelerated fractionation regimens, designed to improve tumour control [9], [10], [11], and has been implicated in causing ‘late’ toxicity [12], [13], [14], [15].

Heterogeneous dose distributions are delivered to the mucosa with intensity-modulated radiotherapy (IMRT), allowing the oral mucosa to be partially spared [16]. However, the development and grade of mucositis is challenging to predict. If a relationship between the dose distribution and toxicity is established, it may be possible to reduce oral mucositis, by means of a treatment planning intervention. Few dose–response studies have been performed for oral mucositis. The complex shape of the mucosal surface, coupled with the fact that it is poorly visualised on computed tomography (CT) are challenges to accurate delineation. Previous oral mucosa dose–response studies [16], [17], [18] have reduced the complex shape of the oral mucosa surfaces to a solid oral cavity volume. Clinical experience suggests that dose–area effects may influence mucositis severity. Characterising the dose delivered to the surfaces of the oral mucosa is necessary to properly study the dose–response relationship. A method of obtaining the dose distribution delivered to the oral mucosa surfaces would, therefore, represent an important advance in attempts to model and, thereby, reduce oral mucositis.

The primary aim of this study was to develop CT-based delineation guidelines to contour the surfaces of the oral mucosa in an anatomically realistic manner. The secondary aim was to establish the worth of the new contouring approach for dose–response studies by assessing the magnitude of the differences in dose metrics extracted from treatment plans using the new, more realistic approach, and previously used contouring techniques.

## Materials and methods

### Patients

Treatment plans of 11 head and neck radiotherapy patients treated at our institution between 2006 and 2013 were included in the study. The patients had oral cavity contours (OCC) generated using our previous method [17] (based on the oral mucosa definition described by Eisbruch et al. [19]), and these were used for comparison of the dose distributions extracted using our new technique for obtaining mucosal surface contours (MSC). This cohort incorporated a range of primary disease sites (oropharynx, hypopharynx and nasopharynx), two different CT scanners and included edentulous and dentate patients, eight of whom had dental implants.

### Structure definition

**Fig. 1:**
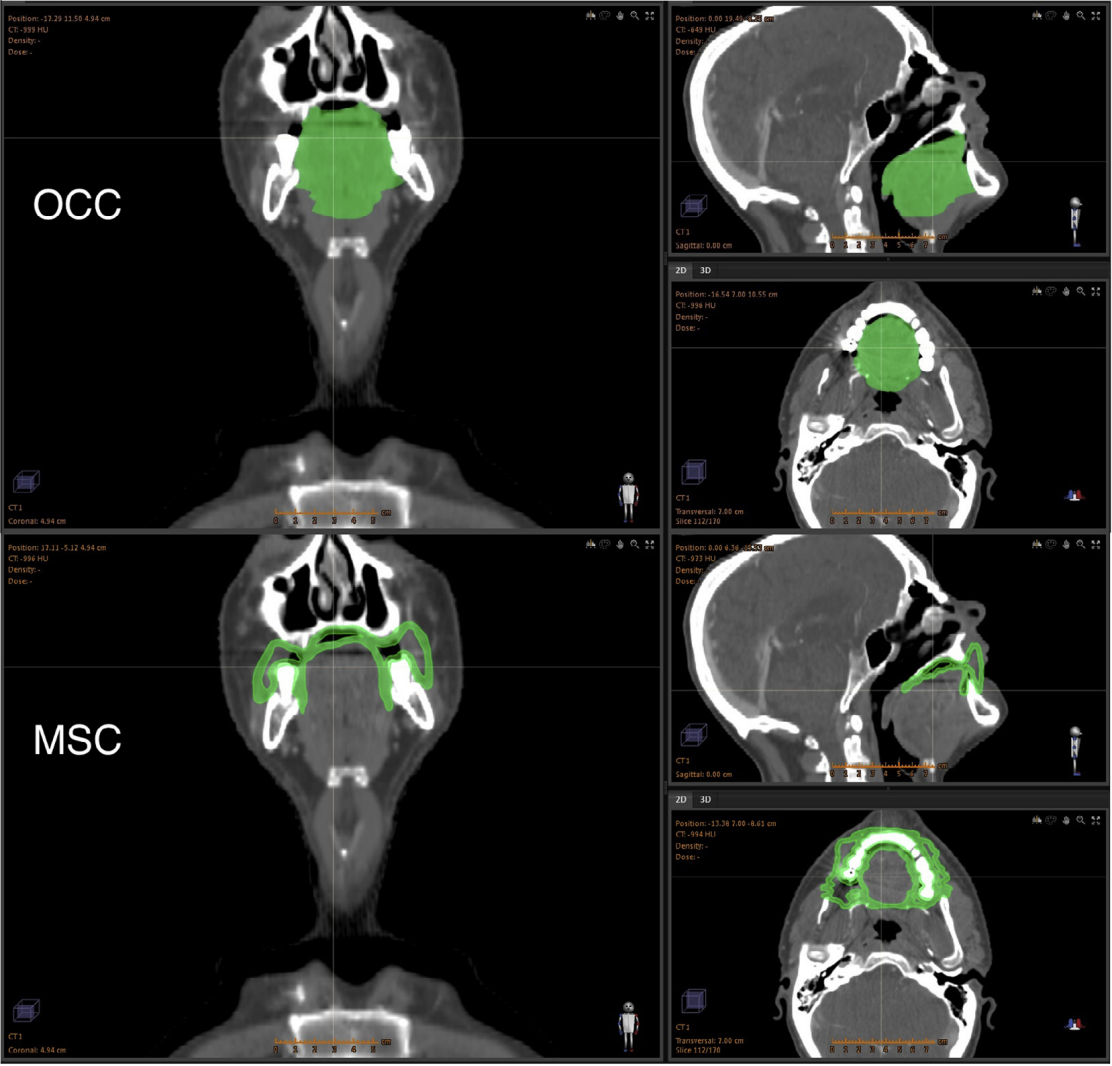
CT scan of a head and neck radiotherapy patient with the OCC (top) and MSC (bottom) shown in green. The OCC representation reduces the complex shape of the mucosal wall to a simplified solid volume, whereas the MSC structure is represented as a mucosal surface and, as such, represents a more anatomically realistic description of the oral mucosa.

The MSC were defined as a 3 mm thick wall of tissue based on work by Ueno et al. measuring the oral mucosal thickness at multiple sites in five cadavers using a reamer method [20]. They measured a mean thickness of 3.12 ± 1.43 mm. The outlined MSC included the following surfaces: buccal mucosa, buccal gingiva, gingiva proper, lingual gingiva, lingual frenulum, alveolar mucosa, labial mucosa, labial gingiva, labial frenulum, mucosal surface of the floor of mouth, mucosal surface of the tongue anterior to the terminal sulcus, and the mucosal surface of the hard palate. The superior extent was defined to be the superior border of the labial mucosa of the upper lip anteriorly, the roof of the palate posteriorly and the superior extent of the buccal mucosa laterally. The inferior extent was formed by the inferior border of the labial mucosa of the lower lip anteriorly, the surface of the tongue posteriorly and the inferior extents of the floor of mouth mucosa and buccal mucosa laterally. The lateral extents of the buccal mucosa formed the lateral borders. The anterior border followed the alveolar mucosa and the posterior extent of the hard palate formed the posterior border (Fig. 1).

### Structure delineation

Structure delineation was performed, by a head and neck radiation oncologist (LW), using the RayStation version 4.0 treatment planning system (RaySearch Laboratories AB, Stockholm, Sweden). The majority of contouring was performed on coronal slices as the main axis of the structure runs in the anterior–posterior direction. Using the coronal plane is beneficial in being able to capture the arch of the palate and the superior, inferior and lateral extents of the buccal mucosa. Sagittal views were useful in establishing the position of the posterior border and axial views aided in contouring the mucosa of the retromolar trigone. The MSC were initially delineated as a single line and, once complete, expanded to a 3 mm annulus. The delineation process was semi-automated using atlas-based segmentation (described in the Supplementary Material).

### Comparison of geometries

The geometries of the OCC and MSC structures were compared by measuring the volumes of these structures and the volumes of their overlap with the primary planning target volume (PTV). To gain an appreciation of the difference in the surface areas of the OCC and MSC structures, an ‘OCC surface’ was defined as a 3 mm annulus extending inwards from the outer surface of the OCC. The volumes of these ‘surface’ structures were then compared to the MSC volumes.

### Comparison of dose distributions

Comparisons of the dose distributions to the oral mucosa extracted using the MSC method and the OCC method previously used at our institution were performed. Neither set of contours was used for treatment plan optimisation. All doses are quoted as the equivalent dose in 2 Gy fractions with an α/β ratio of 10 [21].

As a result of the sparsity of dose–response studies for oral mucosal toxicity, there is currently a lack of strong evidence on the importance of specific dose–volume parameters for mucositis outcomes. Clinical experience suggests that mucositis is a ‘parallel’ type toxicity. Therefore, mean dose, which incorporates information from every dose level, is a useful descriptor of the dose distribution and so was chosen for quantitative dosimetric comparisons. Maximum dose is included in the Supplementary Material for completeness.

## Results

### Structure delineation

An anatomically realistic delineation of the oral mucosa was achieved in all patients (Fig. 1 shows a representative example case). Eight of the 11 patients had streak artefacts on their CT scans due to dental implants. These increased the uncertainty as to the exact location of the mucosal surface where it was obscured by artefact. Whilst the artefacts greatly hinder mucosal contouring on axial slices, in the coronal and sagittal planes following the shape of the mucosa allowed the position of obscured mucosa to be manually imputed with relative ease. The volumes of the OCC, OCC surface and MSC for each patient, and the overlap volume of the OCC, OCC surface and MSC with the primary PTV are shown in Table 1 in the Supplementary Material.

### Dose comparison

**Fig. 2:**
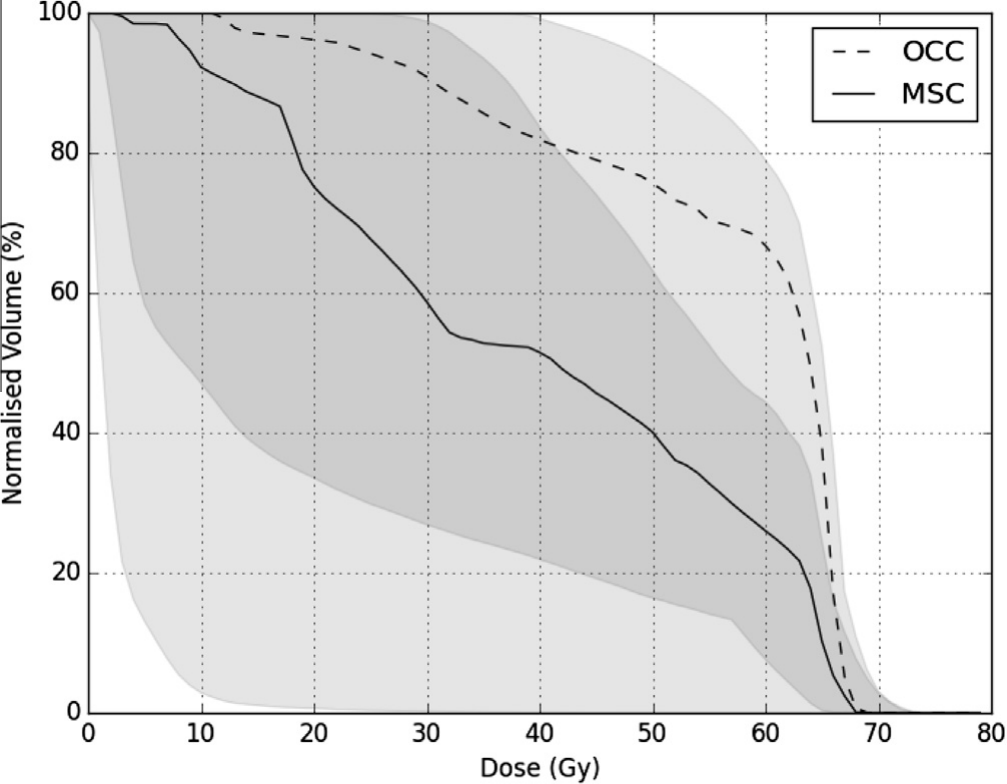
Median dose–volume histograms for the oral mucosa for the 11 patients using the OCC (dashed line) and MSC (solid line) delineation approach. The shaded areas represent the ranges. The darker shaded area shows the overlap between the ranges.

Using the MSC delineation method led to differences in the dose distributions (Fig. 1 in the Supplementary Material shows a representative example case) and dose–volume histograms compared with the OCC method (Fig. 2). The median (and range) of the reduction in the mean mucosal dose when the MSC contours were used was 28.7% (12.8–84.5%) (The individual values for all of the patients are shown in Tables 2 and 3 in the Supplementary Material). The locations of the maximum doses, as obtained using the OCC method, were either in the musculature of the tongue or floor of mouth, within the PTV. These are not included in the MSC volumes.

## Discussion

In this work we have demonstrated that it is possible to delineate the oral mucosa of head and neck radiotherapy patients as an anatomically realistic mucosal surface structure on planning CT scans, rather than as an anatomically unrealistic solid organ.

There is a large difference in the geometry of the MSC, compared with the OCC (Fig. 1 and Supplementary Material Table 1). The majority of the volume difference represents the volume of the musculature of the tongue and floor of mouth, which are not included in the MSC. The volumes of the MSC structures are greater than those of the corresponding OCC surface structures in all cases, indicating that the MSC structures exhibit larger surface areas. These volume differences translate into large differences in the characterisations of the oral mucosal dose distributions, particularly at intermediate dose levels (Fig. 2 and Supplementary Material Fig. 1).

Reductions in the mean mucosal doses with the MSC method were demonstrated for all patients, when compared with the OCC method. The differences in these dose metrics between the two contouring techniques are due to greater overlap between the primary PTV and the OCC than the MSC (Table 1 in the Supplementary Material). The other major source of differences in the dose metrics is due to the MSC extending laterally, away from the high dose region (which tends to be medially concentrated for the primary disease sites of patients in the study), relative to the OCC, which are centrally confined. The findings of Narayan et al. [22] give us reason to believe that the magnitudes of the differences in the mean doses found between the two oral mucosa outlining methods would be clinically significant in terms of predicting mucosal toxicity outcomes and, therefore, would also influence radiotherapy planning interventions aimed at reducing mucosal toxicity.

Few attempts to extract dose distributions for the oral mucosa have previously been carried out. Sanguineti et al. defined the oral mucosa as a single solid volume encompassing the oral cavity (including buccal mucosa), oropharynx and hypopharynx [16]. Werbrouck et al. contoured the oral cavity volume, including the buccal mucosa, but excluded the air within the cavity [18]. van de Water et al. studied the dose–response of the oral mucosa for xerostomia [23], rather than mucositis. They divided the oral mucosa into smaller substructures in an attempt to establish which substructures, if any, are most radiosensitive for xerostomia-related endpoints. A limitation of this approach is that it does not allow for dose–area effects of the entire mucosa to be assessed robustly.

Analysis of the treatment plans showed that the maximum doses reported using the OCC method were located within the musculature of the tongue or the floor of mouth in regions close to or within the PTV. Therefore, no treatment planning intervention to reduce doses to these regions would be possible without also compromising local disease control. However, most of the oral mucosa surface lies outside the PTV and, hence, it should be possible to achieve clinically feasible dose-sparing across at least some of these regions. To this end, we suggest that characterising the oral mucosa dose distribution using our new anatomically realistic approach would provide meaningful dose information that would allow for the modification of treatment plans in an attempt to reduce mucosal toxicity.

We recognise that, within many radiotherapy treatment planning systems, it is not currently possible to perform contouring in the coronal and sagittal planes or to construct an atlas for semi-automated structure segmentation. Whilst contouring solely in the axial plane would be possible by viewing the axial contours in the coronal and sagittal planes before editing them in the axial plane, this would be inefficient. We recommend that treatment planning system vendors make these features available in the future, as they are necessary to accurately contour structures with complex shapes.

The method we have developed for oral mucosal surface outlining provides a means of obtaining a more anatomically representative characterisation of the dose distribution delivered to the oral mucosa surfaces than has previously been achieved. This will enable the acquisition of higher quality dosimetric data for use in future studies of oral mucositis dose–response.

## Conflict of interest statement

We wish to confirm that there are no known conflicts of interest associated with this publication and there has been no significant financial support for this work that could have influenced its outcome.
