# Supplementary material for: A novel method for delineation of oral mucosa for radiotherapy dose–response studies
Source: Radiother Oncol. 2015 Apr;115(1):63–6. doi: 10.1016/j.radonc.2015.02.020 (PMC4641317; doi:10.1016/j.radonc.2015.02.020)
Supplement: Supplementary Material — Tables and figures. [file mmc1.docx]

**Supplementary Material**

**Automated segmentation**

Atlas-based segmentation (ABS) was used to partially automate the delineation process. The RayStation treatment planning system provides an option for the user to create a custom atlas for any structure they wish to define using data from patients treated at their own institution. A semi-automated segmentation protocol utilising the RayStation ABS module was employed. The RayStation implementation of ABS selects and loads the set of contours from the best matching case from an atlas of sets of contours from previous cases, with the best match selected using a rigid registration algorithm (with multiple resolution levels, six degrees of freedom and correlation coefficient as the similarity measure (personal communication)). The best matching contours are then adapted to the new case using a two-pass deformable image registration (with multiple resolution levels, a free form algorithm constrained by a regularisation term to prevent inverted elements in the final deformation field and correlation coefficient as the similarity measure (personal communication)). An atlas of MSC was created using CT data of patients treated with head and neck radiotherapy by sequentially adding the 11 patients used in this study into the atlas, after the mucosal contouring for that patient was complete. The protocol used was as follows:

1. Run ABS on the new patient.

2. Manually adjust the resulting contours (performed by a head and neck radiation oncologist (LW)).

3. Add the final structure contours to the atlas.

4. Generate a 3 mm annulus (1.5 mm in each direction from the contour line) from the structure contours to create the MSC with a 3 mm thick wall.

A 1.5 mm uniform expansion around the contour line, rather than 3 mm into the ‘substance’ of the structure, was performed because there is uncertainty in positioning the contour line exactly at the epithelial surface of the mucosa due to lack of contrast and partial volume effect. The annular structure is not saved to the atlas, as the manual contouring process is easier when using a single line to represent the mucosal surface, rather than a 3 mm thick annulus.

Other groups have previously assessed the accuracy and time gain achieved by ABS tools in segmenting head and neck organs at risk [1, 2, 3, 4]. These studies found that the segmented structures required manual editing, but substantial time gains were still achieved. The aim of using ABS in our study was to aid manual contouring of a novel structure rather than measure the performance of the RayStation implementation of ABS.


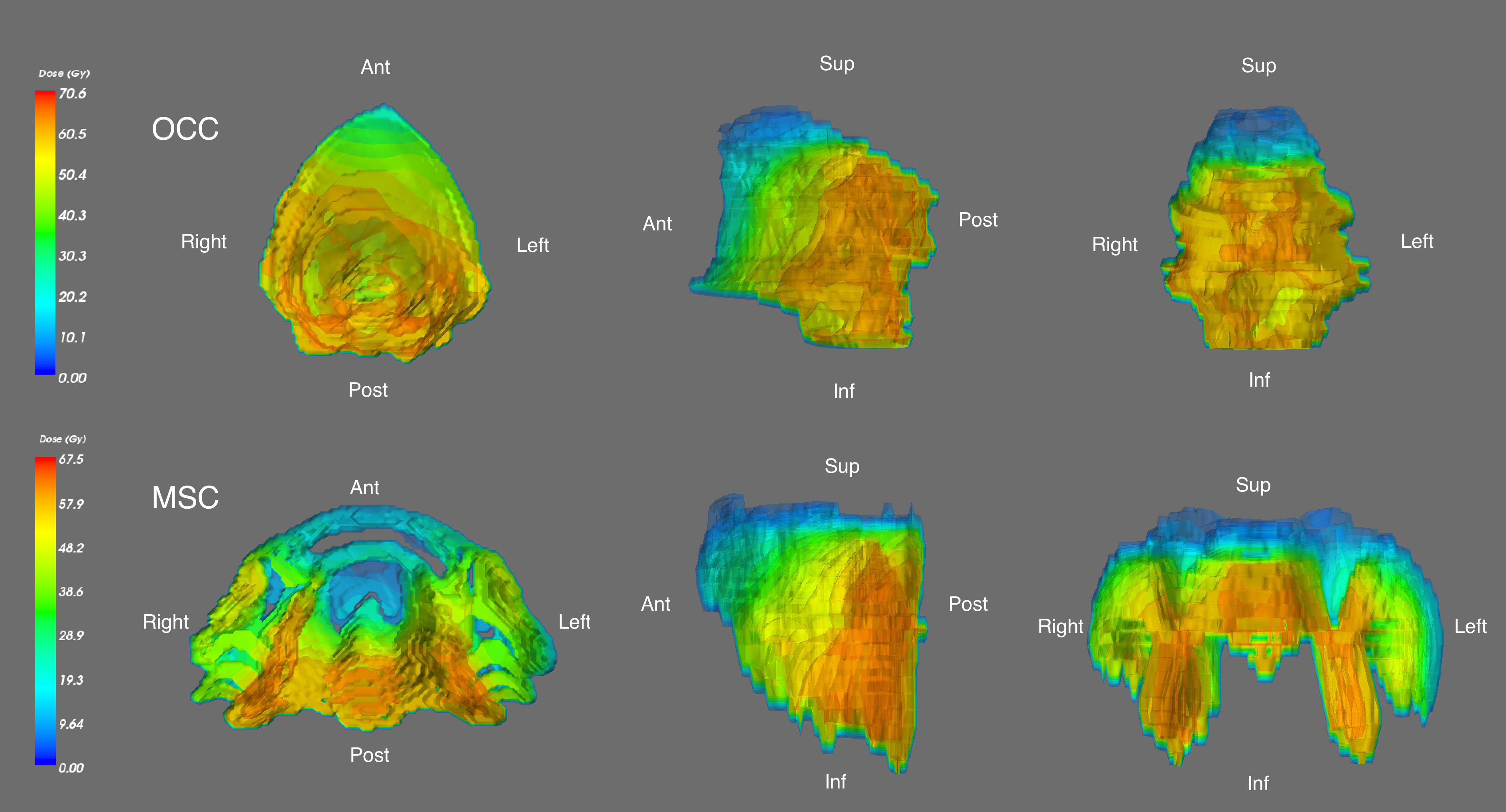


**Fig. 1.** 3D dose maps of the oral mucosa for the same patient using the OCC (top row) and MSC (bottom row) delineation approaches. Three orthogonal views are shown: axial (left column), sagittal (centre column) and coronal (right column).

**Table 1.** Volumes of the previously used oral cavity volume contours (OCC and OCC surface) and the novel oral mucosa surface contours (MSC).

| Patient | Volume (cm^3^) | | | Overlap with PTV (cm^3^ (%)) | | |
| --- | --- | --- | --- | --- | --- | --- |
|  | OCC | OCC Surface | MSC | OCC | OCC Surface | MSC |
| 1 | 124.8 | 50.5 | 68.4 | 55.4 (44.4) | 27.0 (53.5) | 13.2 (19.3) |
| 2 | 117.0 | 37.8 | 66.7 | 3.0 (2.5) | 2.2 (5.8) | 1.3 (2.0) |
| 3 | 90.3 | 30.2 | 40.9 | 16.0 (17.7) | 6.8 (22.5) | 0.9 (2.2) |
| 4 | 117.5 | 41.6 | 62.0 | 42.9 (36.5) | 14.5 (34.9) | 4.6 (7.4) |
| 5 | 150.4 | 45.3 | 69.2 | 83.9 (55.8) | 20.0 (44.2) | 6.2 (8.9) |
| 6 | 94.6 | 32.4 | 53.8 | 47.4 (50.1) | 13.6 (42.0) | 5.3 (9.9) |
| 7 | 93.1 | 34.4 | 55.9 | 55.7 (59.8) | 19.8 (57.6) | 11.0 (19.7) |
| 8 | 108.4 | 34.8 | 60.5 | 8.1 (7.5) | 3.8 (10.9) | 0.0 (0.0) |
| 9 | 109.6 | 36.1 | 56.8 | 85.4 (77.9) | 24.9 (69.0) | 18.8 (33.1) |
| 10 | 127.5 | 38.1 | 48.5 | 69.0 (54.1) | 17.5 (45.9) | 6.2 (12.8) |
| 11 | 117.4 | 37.0 | 46.0 | 68.2 (58.1) | 19.6 (53.0) | 11.2 (24.3) |

The percentage difference in the volumes of the two different sets of contours is calculated for each patient. A negative difference represents a decrease in the volume when going from the previously used to the new delineation technique.

**Table 2.** Calculated mean doses to the previously used oral cavity volume contours and the novel oral mucosa contours.

| Patient | Mean Dose (Gy) | | Difference in Mean Dose (%) |
| --- | --- | --- | --- |
|  | OCC | MSC |  |
| 1 | 56.4 | 42.4 | -24.8 |
| 2 | 41.0 | 24.1 | -41.2 |
| 3 | 46.5 | 29.1 | -37.4 |
| 4 | 58.3 | 43.0 | -26.2 |
| 5 | 57.6 | 37.6 | -34.7 |
| 6 | 51.7 | 30.0 | -42.0 |
| 7 | 62.3 | 48.4 | -22.3 |
| 8 | 20.7 | 3.2 | -84.5 |
| 9 | 63.2 | 55.1 | -12.8 |
| 10 | 52.2 | 37.2 | -28.7 |
| 11 | 61.5 | 49.3 | -19.8 |

The percentage difference in the mean dose obtained using the two different sets of contours is calculated for each patient. A negative difference represents a decrease in the mean dose when going from the previously used to the new delineation technique.

**Table 3.** Calculated maximum doses to the previously used oral cavity volume contours and the novel oral mucosa contours.

| Patient | Maximum Dose (Gy) | | Difference in Maximum Dose (%) |
| --- | --- | --- | --- |
|  | OCC | MSC |  |
| 1 | 70.6 | 67.5 | -4.4 |
| 2 | 67.4 | 67.8 | 0.6 |
| 3 | 71.6 | 67.2 | -6.1 |
| 4 | 68.4 | 68.1 | -0.4 |
| 5 | 72.6 | 72.4 | -0.3 |
| 6 | 70.2 | 69.6 | -0.9 |
| 7 | 75.1 | 74.5 | -0.8 |
| 8 | 70.6 | 51.1 | -27.6 |
| 9 | 69.8 | 69.8 | 0.0 |
| 10 | 71.1 | 69.1 | -2.8 |
| 11 | 70.5 | 69.9 | -0.9 |

The percentage difference in the maximum dose obtained using the two different sets of contours is calculated for each patient. A negative difference represents a decrease in the maximum dose when going from the previously used to the new delineation technique.

**References**

[1] Zhang T, Chi Y, Meldolesi E, Yan D. Automatic delineation of on-line head-and-neck computed tomography images: toward on-line adaptive radiotherapy. Int J Radiat Oncol Biol Phys 2007;68:522-30.

[2] Sims R, Isambert A, Grégoire V et al. A pre-clinical assessment of an atlas-based automatic segmentation tool for the head and neck. Radiother Oncol 2009;93:474-8.

[3] Teguh DN, Levendag PC, Voet PWJ et al. Clinical validation of atlas-based auto-segmentation of multiple target volumes and normal tissue (swallowing/mastication) structures in the head and neck. Int J Radiat Oncol Biol Phys 2011;81:950-7.

[4] Daisne J-F, Blumhofer A. Atlas-based automatic segmentation of head and neck organs at risk and nodal target volumes: a clinical validation. Radiat Oncol 2013;8:154.
